# Latin American Youths’ Migration Journeys and Settlement in the Tarapacá Region in Chile: Implications for Sexual and Reproductive Health

**DOI:** 10.3390/ijerph192013583

**Published:** 2022-10-20

**Authors:** Alexandra Obach, Alejandra Carreño, Michelle Sadler

**Affiliations:** 1 Programa de Estudios Sociales en Salud, Instituto de Ciencias e Innovación en Medicina, Facultad de Medicina Clínica Alemana, Universidad del Desarrollo, Santiago 7610658, Chile; 2 Departamento de Historia y Ciencias Sociales, Facultad de Artes Liberales, Universidad Adolfo Ibáñez, Santiago 7941169, Chile; 3 Women’s and Gender Studies Program, University of Haifa, Haifa 3498838, Israel

**Keywords:** international migrants, adolescent, sexual health, reproductive health services

## Abstract

The sexual and reproductive health of young migrants has not been sufficiently addressed in mobility studies. In this article, we dwell on some aspects of this issue in the migration process of Latin American youth. We conducted a qualitative study in the region of Tarapacá, Chile, carrying out in-depth interviews with key informants, health staff and young migrants between 18 and 25 years old. The results show some motivations to migrate related to sexual and reproductive health: young pregnant women, LGBTQI+ and HIV-positive people seeking access to health care and social contexts of reduced gender discrimination. During the migration process, young people are exposed to various kinds of sexual violence, and in their settlement in Chile, to situations of racism, stigma and discrimination in society as a whole and in access to and during sexual and reproductive health care. Health care for young migrants is mainly focused on maternal care and reproductive issues, while sexual health as a whole is disregarded. We argue that sexual health must be addressed as a central dimension of the lived experiences of young migrants, and that the social, cultural and structural factors that undermine their sexual and reproductive health must be addressed in order to provide culturally competent health services.

## 1. Introduction

The economic growth and political stability that has characterized Chile since the 1990s positioned it as one of the most prosperous countries in Latin America and the Caribbean [1]. This generated a progressive increase in the migrant population arriving from other countries of the region, referred to as South–South migration: communities arriving from Andean countries such as Peru, Bolivia and Ecuador were succeeded by those from Colombia, the Dominican Republic and Haiti, and from Venezuela in recent years [2]. The differences regarding international migrants’ countries and cultures of origin make the experiences around the migratory process highly heterogeneous. As Chile, Peru and Bolivia are bordering nations, human movement between their territories has been a historical constant. This occurs mainly in the north of Chile, where there is permanent trade and service exchange between the three nations. In addition, they share social and cultural aspects that bring them close. A different case are the much more recent migratory flows from countries such as Colombia, the Dominican Republic, Ecuador and Venezuela, from 2005 onwards, where although a common language is shared-Spanish-there are bigger cultural differences. In the cases of Venezuela and Haiti, due to long lasting contexts of social conflict, economic crisis and poverty, many migrants are seeking refuge and asylum [3,4]. Moreover, most migrants from Haiti speak Creole, which adds linguistic barriers. In addition, from these last four countries, the afro-descendant population has added a new scenario in Chile in terms of corporalities and racial hierarchies, given that there was almost no afro-descendant population before this migratory wave.

Despite the heterogeneity of the migrant populations arriving in Chile, those living in vulnerable social conditions are exposed to health risks and human rights violations during the migration process, which can negatively influence their health conditions. In addition, many groups of international migrants experience structural vulnerability during mobility because of limited access to health care, both in transit and upon arrival in the receiving country. This is especially frequent in irregular international migrants, refugees, and those migrants who live in poverty [4]. In addition, they share the fact of being treated as outsiders by nationals and by migrant communities that have been established for a longer time in Chile and in a regular migratory situation [5]. The latter is reflected in discriminatory and xenophobic actions towards migrant groups, especially those in a situation of greater social vulnerability.

Since the onset of the COVID-19 pandemic, and with the consequent closure of borders, entry to Chile through non-authorized points has increased dramatically, creating an unprecedented humanitarian crisis, and thereby increasing the vulnerability of the migrant groups, especially in the north of the country [6]. Today, around 1,500,000 migrants live in Chile, representing almost 8% of the national population [2]. However, as the recent social crisis that began in 2019 has revealed, behind the apparent economic stability that attracts these Latin American flows, there are important hidden forms of poverty, segregation and inequity in access to fundamental rights, among which is the right to health care [7].

The Chilean health system is organized in a mixed private–public system, in which public services attend 70% of the population, including the most vulnerable social groups and those with the greatest burden of disease [8]. The public sector cares for the majority of the foreign population in the country, through a regulation that protects universal access to health for migrants regardless of their legal status [9]. Though their access to health services should be unrestricted, there are current reports denouncing arbitrary administrative barriers that are encountered when seeking access to biomedical services, in addition to episodes of discrimination and denial of care that particularly affect the population in an irregular situation [10,11]. It is important to highlight that although in this paper we are focusing on the migrant population’s access to biomedical services, biomedicine is not the only health system to which the migrant population resorts. They also seek alternatives in medical pluralism that exist in the border area, which includes indigenous medicines, and complementary and alternative therapies, as well as the added different knowledges and practices in health care that migrants bring with them [9].

Regarding biomedical care, studies show how the health inequities encountered by the most disadvantaged socioeconomic groups are exacerbated within the migrant population [12]. Among them, most health-related research has been carried out with adult women and children, leaving some groups relegated, such as young people. In 2017, 245,861 young migrants (15–29 years old) were residing in the country, representing 33% of the total migrant population [13,14]. This figure may have increased given the large influx of people through non-authorized crossing paths during the COVID-19 crisis, especially because the borders were closed in 2020 as a prevention measure for the spread of the disease [15].

The international literature on the health of young migrants raises the need to pay attention to their sexual and reproductive health (SRH) [16,17,18]. Much of the literature that has addressed these issues in migrant youth has done so by emphasizing a risk approach, which tends to focus on behavioral and cultural aspects, downplaying the relevance of structural and sociopolitical factors that shape the conditions in which they live [19,20]. The most frequent risks described in the literature for this group are lower healthcare utilization and trust; inadequate knowledge of SRH and preventive screening practices [21]; patriarchal values and culturally prescribed gender roles that affect women’s access to contraception and family planning [22]; and greater exposure to engage in transactional sex and have sex while drunk or under the effect of drugs [20]. Gender differences have also been described in these studies, revealing that migrant women tend to have less information, access and freedom to demand the use of condoms than men [19,23]. While an account of these elements is certainly relevant, it is crucial to avoid labeling young migrants as a risk group. Instead, approaches that frame their experiences in the context of the multiple forms of social inequalities they face in host countries should be promoted. Overcoming the “risk approach” in the health of young migrants also implies considering the mobility of these groups as marked by factors that go beyond what is strictly economic [9].

In Latin America, there are few studies regarding the health of adolescent and young migrants [21]. Among the international evidence that has been gathered, we know that SRH approaches for young migrants, as well as those for the general young population, are mainly focused on reproductive health, undermining the importance of sexual health. In fact, the association between women and reproduction continues to be present in most policies that address the health of migrants. In Latin America, evidence shows a significant concentration of studies on maternity, maternal and child health, and pregnancy outcomes in the migrant population [24,25]. Regarding sexuality, studies have focused on HIV management [26], excluding other sexually transmitted infections (STIs). There has also been little attention given to the LGBTQI+ population and that of migrant sex workers, despite the growing presence of these subgroups within migrants [27,28] and the growing evidence of the disadvantages they face in the health care systems [29].

The intersectionality approach in migration studies has highlighted the impact of ethnic and racial components on xenophobia and discrimination against certain groups, such as Afro-descendant people. Studies in Chile have shown how Colombian, Ecuadorian and Dominican Afro-descendant women came to represent an important part of the sexual trade in border and industrial areas in just a few years [28,30] since various structural processes of exclusion hinder their employment in other areas [31].

These background elements are relevant for the study of the migratory experiences of young people and their access to formal health systems in the receiving countries. In this article, we are interested in dwelling on some aspects of SRH in the migration process of Latin American youth; during the journey, in their entry and settlement in Chile, as well as in their health care seeking experiences.

## 2. Materials and Methods

### 2.1. Study Design and Setting

We conducted a qualitative study (Fondecyt Iniciación #11190701) with a descriptive approach, seeking to understand how people give meaning to their social environment and how they interpret it [32]. For such purposes, we carried out in-depth individual interviews with key informants, health professionals and young migrants (aged 18–25). The study was conducted in the Tarapacá Region in Chile, located in the extreme north of the country, which limits to the east with Bolivia. The region has the fourth highest number of migrant inhabitants in the country. Fieldwork was carried out in the two districts with higher migrant residents within the region: Iquique, where there were an estimated 44,304 foreign residents in 2019, and Alto Hospicio, with 17,937 [33].

### 2.2. Operational Definitions

In this study, we refer to “migrants” as persons who at the time of fieldwork were living in Chile, specifically in the Tarapacá Region, and who were born in other Latin American countries. We follow the definition of migrants given by the International Organization for Migration, referring to “a person who moves into a country other than that of his or her nationality or usual residence, so that the country of destination effectively becomes his or her new country of usual residence” [34].

### 2.3. Participant Selection and Recruitment

Three categories of respondents were recruited: key informants, health staff working in SRH services, and young migrants of different Latin American nationalities between the ages of 18 and 25.

Key informants were recruited purposively given their expertise in the topics of SRH services, health of migrants and/or young people. We recruited national and local health authorities, academics and members of community organizations and NGOs to gain a broad understanding of the challenges young migrant people face when trying to access SRH services. The final sample included eight key informants (two from academia, two health authorities and four members of civil organizations). Health staff working in public health services in areas of SRH, migration and youth were recruited to participate in interviews. The first contacts were given by local key informants, and some of the following contacts were identified by snowball sampling of interviewees. Others were approached directly in Family Health Centers (CESFAMs) settled in districts with high migrant populations. The final sample included 10 health staff working in CESFAMs settled in districts with high migrant populations (five midwives, two social workers, one physician, one psychologist and one nutritionist).

Regarding the young migrant individuals, eligible participants were 18–25 years old, who were born in a Latin American country other than Chile, spoke Spanish, had migrated to the country at least three months before the interview, and were living in vulnerable social conditions. Recruitment occurred through purposive and snowball sampling. The selection of cases was intended to include young migrant people living in urban and rural areas, from different nationalities and who identified as heterosexual and LGTBQI+, in order to understand how various social markers interact with perceptions of SRH and health care seeking behaviors. The final sample included 17 young migrants: six persons who identified themselves as women, six as men and five as LGBTQI+. Six of the young migrant participants were Ecuadorian, five Colombian, five Venezuelan, and one Peruvian. Five of them were 18–19 years old, seven were 20–22 years old and five were 23–24 years old. All participants were interviewed individually, and they were all living in the Tarapacá Region at the time of the interviews.

### 2.4. Data Collection

Data collection was programed to begin in March 2020, but, due to the COVID-19 pandemic, it was postponed for one year. It resumed in March 2021 and was carried out until May 2022, during which period quarantines and mobility restrictions still affected the possibility of carrying out face-to-face encounters. Thus, interviews were carried out in different modalities depending on the COVID-19 situation at the time of the fieldwork. Data collection was carried out first with key informants, then with health personnel and finally with young people. This involved the gradual lifting of COVID-19 restrictions, in such a way that most key informants were interviewed through Zoom, health staff were interviewed through Zoom and face-to-face, and all young migrants were interviewed face-to-face. Face-to-face interviews were conducted at health centers in the case of health staff, and in physical locations chosen by participants, which allowed privacy, in the case of young migrants.

Interviews with key informants and health staff were moderated by researchers/authors AO, AC and MS, and those with young migrants by a local researcher who lives in the Tarapacá Region and has broad experience working with the migrant population. Researchers AO, AC and MS have extensive previous experience in conducting qualitative research, including expertise in the SRH of young people and the migrant population, and in assessing public policies on these matters. Interviews extended from 40 to 80 min and were all conducted in Spanish; they were carried out within each category of participants until data saturation was achieved. All participants signed an informed consent prior to the interview.

In-depth interviews were used to collect information because they help to obtain profound information on perceptions of SRH and about health care seeking experiences. Interview guides were constructed by the research team based on the study objectives and including aspects derived from an updated literature review. Two interview guides were constructed: one for key informants and health staff, and another for young migrants. They were tested through pilot interviews with each of the social actors addressed by the study and improved based on said pilot, and they were refined throughout the study. The questions were asked with an open approach, which allowed participants to expand freely on their perceptions and experiences of each topic addressed. The following topics were covered in interviews with key informants and health staff, referring to young migrants: (i) governmental and regional/local approaches, policies and programs for SRH; (ii) SRH needs; (iii) gender and sexual identities and practices; (iv) health care seeking behaviors and health itineraries; (v) barriers and facilitators to access health services; and (vi) health staff perceptions of young migrants. With young migrants, the following topics were covered: (i) the migration process and life in Chile; (ii) gender and sexual identities; (iii) health biography, health care seeking behaviors and experiences in the country of origin and in Chile; and (iv) met and unmet needs in SRH.

### 2.5. Qualitative Data Analysis

In-depth interviews were audio recorded and transcribed verbatim using Microsoft Word, and each transcript was checked for accuracy against the original recording by the researcher who had moderated the encounter. The transcripts were analyzed using thematic analysis, a qualitative method that enables thematic patterns to be identified from the collected data [35], with the support of software ATLAS TI8. All researchers/authors conducted the analysis based on the following steps: familiarization of the transcripts by reading and re-reading the texts; identification of preliminary codes by noting emerging issues from the data; grouping related codes in clusters; creating a code book with main and subordinate codes; and identifying patterns across interviews. During each step, the researchers compared notes and resolved disagreements by discussion until consensus was reached. The verbatim quotations selected for this article were translated by a professional translator and checked by authors to verify the original meaning was maintained. In order to assess the rigor of the study, triangulation of responses from several participants (key informants, health staff and young migrants) was carried out [36].

### 2.6. Ethical Considerations

The study was approved by the Ethics Committee of Universidad del Desarrollo (IRB 2019-22), which is officially registered by the US Office for Human Research Protections. Participants received information about the objectives and procedures of the study orally and in writing, detailing the benefits and potential risks of the study, study procedures, and issues relating to audio recording, storage and confidentiality. All participants signed an informed consent form before interviews were carried out. Confidentiality and anonymity were ensured by replacing names with interview codes and pseudonyms in the transcripts and publications and by deleting personal information from the transcripts that could identify participants. Data have only been accessed by researchers directly involved in the study and stored securely in a database. Audio recordings were discarded after transcription.

A response protocol was designed in case participants experienced emotional stress during the research process, detailing containment measures for the research team to apply, and guaranteeing subsequent referral to a psychologist and to a range of health services. There was no need to activate such a protocol.

## 3. Results

### 3.1. Description of Results

The results are presented following the migratory process of young people: first, we highlight motivations to migrate; then we show the SRH risks faced during the migration process; third, we report the process of settlement in Chile, focusing on experiences of stigma and discrimination; and finally, we describe some issues regarding young migrants’ access to SRH services in Chile. Table 1 summarizes the main study findings.

### 3.2. Motivations to Migrate

Young people interviewed explain that the decision to migrate could have been taken by their parents or by themselves; all cases were motivated by a need to improve their quality of life. Although economic reasons and political instability were the most frequently mentioned, other important reasons related to SRH appear just as relevant, which can be especially important for some individuals who decided to migrate on their own. Regarding these latter motivations, we identify three main categories: (i) young pregnant women seeking better pregnancy and childbirth care than in their home countries; (ii) LGBTQI+ people looking for social contexts of greater acceptance of gender and sexual diversity; (iii) and HIV-positive people seeking access to treatment. We will focus on these, given they are relevant for the objectives of this work.

Regarding the first category, the interviewed health workers state that the greatest demand for health care by migrants corresponds to pregnancy controls. They affirm that, in recent years, young pregnant women entering the country have increased significantly in the Tarapacá Region, many with very advanced pregnancies and not having had any gestational controls before:

*In Colchane, pregnant women come from Bolivia to Chile and arrive with pregnancies of 32, 36, 38 weeks, so there is no time to do all the necessary exams. The same thing happens in Iquique, they are arriving very late in their pregnancies*.

(Health Staff, physician)

*At least 55–60% of our pregnant women in the region are foreigners*.

(Health Staff, midwife)

Regarding the LGBTQI+ community, when deciding to migrate, Chile appears as an option given it is considered one of the most progressive countries in the region in terms of sexual and gender diversity rights. For this reason, the country has become a benchmark for LGBTQI+ communities, who migrate with the expectation of being able to display their identities more freely than in their countries of origin and thus materialize the life projects they have not been able to carry out. Young LGBTQI+ persons interviewed express this idea clearly:

*In Chile I am calmer, not having to hide or lie. Here it is acceptable for two men to go down the street holding hands, while there [Ecuador], we cannot even sit in parks because they would immediately start throwing stones at us, discriminating against us, insulting us, attacking us. Here you do see that, but not that much, much less than in Ecuador*.

(Migrant, Ecuadorian, man, 21)

Key informants interviewed agree with the statement of young migrants about Chile being perceived by the LGBTQI+ communities in Latin America as a progressive society, that welcomes gender-based diversities and that favors the rights of these groups.

*There is this idea in the LGBT community that Chile is a paradise of rights*.

(Key Informant, member of a migrant organization)

*Chile is one of the countries in the region that, along with Argentina, has made progress on establishing rights for sexual dissidents. In terms of trans issues, in addition, the trans community in Chile is a very organized community, which has even increased political leadership today, so there is a reference to coming to Chile, as “I can be safe in Chile”*.

(Key Informant, member of a migrant organization)

Key informants also express that the repression experienced by sexually diverse communities in some Latin American countries motivated them to migrate:

*We have realized that at some point trans people or dissidents say, “I have to migrate because of my condition”*.

(Key Informant, member of a migrant organization)

Within the trans community, according to the key informants, this perception is exacerbated because the visibility and social participation of the Chilean trans community is greater than in the rest of the region. Moreover, their main health needs relate to access to hormone therapy and surgeries, and Chile is perceived as a territory in which they can access these treatments without major barriers. A transgender activist interviewed explains:

*When I started looking for a country, and I am also talking about other trans friends, the questions were: “Where can I take hormones, where can I have surgery, where can I change my name?” And Chile had health regulations and public hospitals which were doing so, and I thought: “I want to go to there, I want to be able to live, I want to be able to breathe”. That is what the right to migrate means, the intrinsic sensation of migrating, of finally feeling that one can reach his life outside his border*.

(Key Informant, member of a migrant organization)

This perception of Chile is attractive for the LGBTQI+ community, especially for those who suffer discrimination and even violence in their countries of origin due to their sexual/gender identity. According to key informants, in countries such as Venezuela, characterized by a heavily patriarchal and sexist culture, many transgender persons have experienced somebody of their community being killed because of their gender identity.

*… Among the many reasons that they have had to migrate, I think this is the main reason*.

(Key Informant, member of a migrant organization)

They also point out that access to free health treatments for HIV is a key reason for young HIV-positive people to migrate to Chile, given the country has a policy of free antiretroviral therapy:

*In Chile there is greater access, treatment is guaranteed for all people, even if they are tourists, whoever is in Chile can have access to HIV treatment. Other countries do not have that possibility because they do not have financial sustainability to buy the drugs. And that makes us attractive in terms of a country to come to if you need to be in treatment. The epidemiological reports of recent years strongly mark the trend that many people from other countries in the region are arriving to look for medicines. Some come and go, but many stay*.

(Key Informant, academia—expert on HIV and migration)

*Most of the migrant HIV-positive population from the region, and especially Venezuela, come to Chile because they can access their medications here*.

(Key Informant, member of a migrant organization)

*In Venezuela, HIV-positive people have to flee because there is no access to antiretrovirals. Obviously, they need urgent protection, so it’s not a political issue, it’s a humanitarian issue, that one flees from a context of discrimination and seeks protection*.

(Key Informant, regional health authority)

### 3.3. Sexual and Reproductive Health Risks during the Migration Process

Some of the young migrants participating in the study arrived in Chile years ago, directly from their countries of origin, while others have entered in recent years or months. Many of this last group have been traveling on foot from various South American countries such as Venezuela, Colombia, Ecuador, Peru and Bolivia. Some young people make these journeys with their families of origin (parents, siblings, uncles, etc.), with their own newly formed families (partners and children), in groups of friends or, finally, some travel alone. During the migratory process, many young people settle for variable times in the countries through which they transit, where they do sporadic work to raise money and can thus continue their trip. All the stories show the hardship of the migration process, and individuals facing a series of obstacles of various kinds:

*It has not been easy. I have been outside my country for eight years, it has not been easy. First, I left from Venezuela to Colombia, from Colombia to Ecuador, from Ecuador to Peru, then Bolivia, and here, seven months. I did not have a clear destination, I had nobody to receive me, nor did I know the way, but I did it and now I am here in Chile. I have worked in construction, as a waiter. When I left my country I left alone, without money, without any resource, with nothing*.

(Migrant, Venezuelan, man, 22)

Engaging in commercial sex work is one of the strategies that young woman engage in during the migratory process, in order to survive, as a young Venezuelan woman describes:

*[In Colombia] There I had to work as a prostitute. I had to work in a brothel (…) I lasted about a month working there, it was very hard*.

(Migrant, Venezuelan, woman, 19)

One of the themes that appears recurrently in young people’s stories relates to exposure to various situations of sexual violence during the migration process. These experiences occur mainly during border crossings, where, on many occasions, transportation must be paid through sexual favors, either to the truck drivers who transport them, or to the gangs that help them to enter irregularly to countries in the region. These risk situations affect both women and men:

*To be honest, I lived it *[trading sex for transport and food]*. At that moment when my sister got seriously ill, I experienced it with the man who took us from Lima to Tacna. He never stopped taking care of my sister, he always gave us food... Necessity obliges…*

(Migrant, Venezuelan, woman, 19)

*I have heard that too often, sexual abuse. For example, truckers are predators … “I’ll take you, as long as…”. That, of course, for both men and women. Or, for example, it happened to my wife too, she used to ride up in the front and fall asleep, tired from the road, and they would touch her breasts, her private parts. Things like that. That’s where it starts. And in men too, they offer money, things like that*.

(Migrant, Venezuelan, man, 24)

According to key informants interviewed, although everyone is identified as a potential victim of sexual assault, young women and the LGBTQI+ population are at greatest risk. For this reason, they explain, in some cases trans women choose to make their migratory journey with a male identity as a means of protection:

*When the trans community makes the trip from Venezuela, when it is trans woman who have already started their transition process, they prefer to do the journey dressed as men, because they will be less likely to be raped or groped in buses and other means of transport*.

(Key Informant, member of a migrant organization)

Key informants, in line with the testimonies of young migrants, report that other forms of violence experienced during the journey by young migrants to Chile relate to exposure to sexual health risks as survival strategies, such as engaging in sex work to buy food and basic subsistence items. A participant, belonging to a pro-migrant organization, states that, although all migrants are potentially exposed to these situations, there are certain groups at higher risk, mainly LGBTQI+ people who face situations of greater vulnerability:

*Violence is often related to survival strategies. Especially on the trip, that can be quite long; weeks, months or even years. Many women have had to engage in commercial sex in order to feed themselves. And of course, if you have bad luck there is a possibility of being attacked, raped. Not only women, but also the case of a gay boy who told me about a gang rape that he experienced on the trip*.

(Key Informant, member of a migrant organization)

*[During the migration process] The trans population, the gay population, the lesbian population, they are much more exposed, they are vulnerable to networks of human trafficking*.

(Key Informant, regional health authority)

Although the migratory trajectories are mainly marked by obstacles, several of the young people interviewed mentioned having had support from various national and international agencies throughout their journeys, which deliver personal care kits at border points, including condoms:

*In all aid points they gave me condoms… in Ecuador and in Peru. They gave kits of shampoo, soap, different personal items and condoms, which was the only thing they gave for us to take care of ourselves*.

(Migrant, Venezuelan, woman, 19)

### 3.4. Settlement in Chile: Experiencing Vulnerabilities

The experiences of entering and settling in Chile vary among the young people interviewed. There are those who entered the country several years ago and are settled in a stable manner in the Tarapacá region and are currently studying or are formally or informally inserted in the labor market. In addition, there are those who have entered Chile through authorized and non-authorized paths in the Tarapacá Region in recent years and months. The latter are in growing numbers since 2020, because of stronger restrictions on entering the country and closure of borders due to the COVID-19 pandemic. High numbers of Venezuelans have been entering the country during this period, a migration characterized by massive entry through unauthorized paths with the consequent situation of irregularity in their migratory status which places them in a situation of high social vulnerability. Entry through unauthorized points implies that people cross the Andes Mountain range on foot from Bolivia, having to endure the desert climate, low temperatures and lack of oxygen, with the extreme consequences of death during the journey, or survival in very poor health conditions.

Once they have entered the country, people begin to move towards the cities. For many, the final destination is Santiago, the capital of Chile. The large numbers of migrants who have entered the northern cities have generated an increase in people living on the streets. There has also been an increase in migrant camps: informal ones set up by migrants themselves, and those set up by local authorities. They are mostly settled in land far from the city and are quite precarious. Those set up by migrants in most cases do not have basic services such as drinking water or electricity.

A transversal element in the settlement process of the young people interviewed are experiences of stigma and discrimination. These experiences happen in public spaces such as the street, and in institutions such as schools and health centers. Although all migrants describe that they have felt discriminated against, how this manifests varies greatly. There is a general feeling that they are competing with the local population for the use of public services, for which they feel mistreated in several aspects of their daily lives. This is reflected in testimonies linked to experiences of discrimination based on skin color, as the following quote exemplifies:

*I have had encounters with people who are discriminatory, who are xenophobic … they called me monkey*.

(Migrant, Peruvian, woman, 19)

The most recent migratory flows that have entered the country, coming from countries such as Venezuela, Colombia, Ecuador and Haiti, have led to new discrimination, related mainly to the racialization of black bodies by the national population:

*When I arrived here, because there were not so many foreigners as dark as I am … I was very discriminated against at school, I had to defend myself fighting because it was the only way … With time one begins to assimilate it, accept it, then it’s not so heavy anymore*.

(Migrant, Colombian, man, 18)

Along with this discrimination, young afro-descendant migrant women describe that they are constantly being asked to engage in commercial sex when they are in public spaces. In other words, there is an association between black bodies and commercial sex work that has a strong impact on the migrant populations of the Caribbean:

*I’m walking and cars pull by, it’s men who offer me money. I say ”no” and they leave. That happens a lot here*.

(Migrant, Colombian, woman, 18)

*Here* [In Chile] *I always listen people saying that Colombian women do it better [sex], that they move it better*.

(Migrant, Colombian, man, 20)

*The intention of* [men] *approaching and proposing something to you is... even if you answer in a friendly way, if you give a bit of kindness, they will talk, they will continue, they want to exceed that limit and invite you to something* (sex)... *It’s not OK, it’s usually adult men to be more precise, older men. Whether I’m walking, whether it’s on transport, older men are always going to insinuate themselves that way, more so if you’re young*.

(Migrant, Ecuadorian, woman, 19)

Furthermore, Venezuelan and Colombian migrants are commonly associated with activities such as drug trafficking and violence linked to it, which is another source of stigma and discrimination:

*I have endured humiliation, I have endured rudeness, heard things that no person would like to hear. Because you are an immigrant, because you are Venezuelan, they immediately treat you as a thief, a murderer. … I know what it’s like to live on the street, I know what it’s like to be attacked because you’re an immigrant. I have had to experience things that I would never have wanted to experience*.

(Migrant, Venezuelan, man, 22)

*You listen that “Colombians should leave”, … “They come here to steal from us” … and one is wearing himself out working to have a decent life or trying to have a decent life*.

(Migrant, Colombian, man, 18)

### 3.5. Health System in Chile: Sexual and Reproductive Health Services

Young people interviewed, especially those who have arrived in the country in the last year, report not understanding how the Chilean health system works and, therefore, not accessing it:

*I was in a shelter when I arrived. I received confusing information about the health centers and didn’t understand if I could access care for free or not*.

(Migrant, Venezuelan, man, 24)

A young migrant, recalls how shortly after arriving in the country, he fell very ill and went to the health center:

*I entered with my passport, I had to pay. At that time, I did not know that care should be free, and I was having the fear that I could be sent back to my country* [deported]. *They do not tell you in the health center that it is free. They attend and that’s it, they don’t give you that option or that basic knowledge. Now I have more knowledge*.

(Migrant, Ecuadorian, women, 19)

As the above quote shows, the main questions that run through the experiences of young people are related to the requirements they need to access health, and whether or not they should pay for said access. All young migrants interviewed who have sought health care report having experienced administrative barriers as obstacles to access the health system, especially those in an irregular situation who do not have a provisional or permanent ID, who can be denied care even though they have the right to access free health services. This is, for example, the case for pregnant women interviewed, who describe how they were denied care or mistreated for their migratory status:

*I’m 35 weeks pregnant, I’m in a camp, and I don’t know what’s going to happen, because since I’m from Ecuador they don’t want to help me, so they haven’t helped me or given me any solution. Approximately two months ago I was able to get a check-up with the doctor, but it was also difficult because twice I had complications to arrive on time and then they wouldn’t accept me. I had to walk the entire beach of Iquique to a Health Center that is very far away, the last time we walked five hours to get there, and they did not attend me because I did not have a temporary ID and I was told I need a provisional ID to receive good care, and that’s the way it is*.

(Migrant, Ecuadorian, woman, 20)

*In the emergency room at the hospital, the cloth that they gave me to cover myself was stained with fresh blood from a woman who had given birth, it was full of blood, it was even slimy, like mucus. I told the doctor and she said I should put it back on, and fast. Then she examined me and told me to wait, sitting in the corridor. I was sitting for three hours. This was like a punishment, it happened when I didn’t have my ID. Now that I have a provisional ID, they treat me a bit better*.

(Migrant, Venezuelan, woman, 20)

Young migrants recognize that the discrimination they experience in society as a whole is reproduced in the health system, situations that generates a distance between them and health services. Those who have sought health services state that, frequently, in interactions with health personnel, they feel as if they are utilizing resources that worsen the quality of the health care received by the local population. Thus, some state they would only seek health services in cases of emergency, in order to avoid being mistreated and even deported:

*Going to the hospital makes me insecure, because of everything I’ve heard … if I got very ill, and my only solution was to go to the hospital and they denied me care, I could die. I do not trust the health system*.

(Migrant, Colombian, woman, 24)

As we have seen, pregnancy and childbirth-related health services are the most sought after by migrant young people, and, although they are grateful for being able to access care, they report frequent experiences of mistreatment. They are aware that women in general can suffer obstetric violence, but that it is especially crude when it comes to young migrant women:

*Now with the pregnancy, they have told me that I have to be very strong because here in Chile they say very ugly things to women*.

(Migrant, Colombian, woman, 21)

*When I went to give birth to my daughter, they treated me badly, I was 17 years old at the time. The midwife was very tough, she told me that if I had liked making a baby, I had to put up with it, and if I made noises or yelled, they would tell me to shut up … I think that it was mostly because of my age and because they were racist*.

(Migrant, Ecuadorian, woman, 24)

Nonetheless, there are also good experiences of care during childbirth, but that are perceived as exceptions, as the following quote exemplifies:

*They treated me wonderfully. They even gave me anesthesia. I remember that doctor very much; she treated me very well, everything was fine. So maybe not all of them are bad*.

(Migrant, Venezuelan, woman, 19)

Regarding the positive aspects of health system, one of the main ones identified by young migrants interviewed is the easy availability of contraceptives and condoms in primary care centers. According to them, this is not so common in their countries of origin, which is why they highlight this aspect of the health system in Chile and appreciate it:

*I actually find it very good. Because, for example, in other countries asking for condoms is frowned upon. For example, in Peru, women have to go with their mother to take care of themselves. Then, as there is no confidence to tell yourmother, “Mom, I want to have* [sex] *my first time”, then some diseases occur or an unwanted pregnancy appears. So, the fact that they give any contraceptive method here is very good, I find that it is very good*.

(Migrant, Peruvian, woman, 19)

*In Ecuador there are many pregnant adolescents, I was one of them, at 14 I got pregnant, and, well, in Quito where I am from there are many pregnant adolescent girls of 14 years, 13 years, it is normal*.

(Migrant, Ecuadorian, woman, 21)

*I have four children, one on the way, and two girls and one boy. They are in Venezuela. I was a father at 14 years old, I had no childhood*.

(Migrant, Venezuelan, man, 22)

Although during the last decade many advances in access to the health system for the migrant population were implemented, several of the health professionals interviewed think that the country’s new migration law has generated significant setbacks in terms of their health guarantees. A midwife commented:

*I feel that there has been a setback with the new immigration law. Before, young migrants received everything, regardless of whether or not they had their regular status, but apparently now there have been more requirements regarding the delivery of identity cards to families with young migrants. There is quite a limitation for these people to register, they are more bureaucratic*.

(Health Staff, midwife)

According to key informants, there is also often misinformation among health workers regarding migrants’ health rights. Therefore, many of the regulations that protect their access to health services may not be respected:

*I get the impression that there is a lack of accurate information in those who have to execute the health policies, who are the health workers of the CESFAM. I think there is little preparation for health staff to give this first information; they give the first answer that occurs to them and if they don’t want to attend migrants, they don’t*.

(Key Informant, member of a migrant organization)

*Information is necessary. We have realized that foreigners think that they must have their ID in order to get access to health care, and this is not the case. In primary care, the passport is enough. Sometimes young people get sick and it’s over a year and they don’t seek care because they don’t know that they have the right*.

(Health Staff, midwife)

Members of pro-migrant organizations interviewed add that health is not a priority for young migrants living in vulnerable contexts, since their priority is survival:

*Health is not a priority, because in the priority scheme it would be to feed myself, stay alive and have a place to sleep for now, so health goes to fourth, fifth place*.

(Key Informant, member of a migrant organization)

This is case for several of the young migrants interviewed, who were struggling to survive in very precarious conditions since their arrival to the country and had not approached health services.

Regarding LGBTQI+ young migrant people, the administrative barrier associated with the immigration status of people also affects access to specific treatments, such as antiretroviral therapy for HIV-positive people. Regarding HIV, the main problem is that when people go to the CESFAM without their provisional ID, they face different barriers to access health guarantees. Thus, they cannot access their antiretroviral therapies. Fortunately, the zero-positive community is an organized community and there are NGOs which facilitate access to tests and to antiretrovirals.

The migratory status also affects transgender people, who report that the Chilean protocols for recognizing their sexual identity are, in many cases, not followed when it comes to migrants without permanent residence:

*I have suffered episodes of discrimination, because I still haven’t been able to change my name and sex in the Chilean documents, despite the fact that I’ve been here for six years and still can’t access the law. There is a gender identity law, but the law is not for migrants, but for those who have permanent residence … I have experienced strong episodes of discrimination because of this*.

(Key Informant, member of a migrant organization)

## 4. Discussion

The results of the study show the various areas in which the migratory experience impacts the SRH of young migrants. Different moments of the migratory process are identified in which the areas of sexuality play a central role in the mobility of young people from Latin America within the region.

The first moment refers to the motivations to migrate. Evidence has shown that within the migrant population, the motivations to migrate are varied and are triggered both by the economic and political contexts of the countries of origin, as well as by individual motivations. In the case of young migrants, the literature reports that the decision to migrate is generally not made by them, but by the adults in the family, who seek better economic and employment opportunities [37]. Other migratory motivators are also identified, related to SRH, especially in young people with autonomy in migratory decision-making. This is the case of young pregnant women, who in many cases migrate seeking access to health care in Chile, given that their care should be guaranteed, regardless of their migratory status [38]. In fact, various studies have shown that in Chile, migrant women use prenatal and gynecological care services more than the local population [39]. The results of the study are consistent with the evidence, since several of the young women interviewed state that coverage in Chile for pregnancy and childbirth is better than in their countries of origin. Even so, they report that the experience in health care for these circumstances is often marked by situations of discrimination, xenophobia and even cases of obstetric violence [38]. In Chile, obstetric violence has been mainly studied in the female population in general, not specifically for migrant groups, so this topic is presented as an important emerging finding that is worth further study [40].

The fact of being able to access health services during pregnancy and childbirth in Chile is received by many interviewed migrant women with a feeling of gratitude, but health staff can perceive them as taking advantage of the system, especially if they have irregular migratory status [38]. The testimonies of young pregnant women refer to various barriers in accessing health care, which have been accentuated by the migration crisis and the closure of borders due to the pandemic in 2020 [15]. Bureaucratic obstacles and the fear of being deported for approaching health services are important barriers for the care of pregnant women in an irregular situation today, barriers that have been previously reported in the literature [41].

Another motivation to migrate identified refers to LGBTQI+ people who decide to migrate given the contexts of homophobia and transphobia in their countries of origin. Various studies have named this phenomenon as “sexile”, referring to the fact that people with a non-heterosexual identity are forced to leave their country of origin [42]. Some people migrate because of the structural and interpersonal violence they experience in their countries, because of the censorship of LGBTI+ activism, public persecution, or the lack of public promotion and recognition policies [27]. Although the migration of LGBTQI+ people has received little attention in the South American literature [27,43], the results of the study give an account of this emerging phenomenon, through experiences of young LGBTQI+ people who have migrated to Chile under the assumption the country would provide them a social context of greater openness towards gender-based dissidence. This is promoted by the fact that the country has a gender identity law, which recognizes the right to gender identity and the rectification of sex and registered name [44]. Despite this and other regulatory advances, the study reveals that in the migratory process, LGBTQI+ people continue to experience situations of discrimination, homophobia and transphobia [45]. Nonetheless, they value positively the greater openness they find in Chile, the possibility of publicly living their gender identity and sharing with a community with a wide range of activist groups, which have been key in advancing rights for sexual minorities.

Finally, we identified a motivation linked to access to treatment for STIs, such as antiretroviral treatment for HIV. Bula and Cuello [43] point out that the lack of antiretroviral drugs for LGBTQI+ people has triggered many gay men, bisexuals and trans women to migrate through Latin American countries. In Chile, antiretroviral therapy is universally accessible and does not exclude the migrant population, which makes the country a focus of attraction for people who require it. Although we identify this as a reason for choosing Chile as a destination, in practice the various barriers young migrants encounter in accessing health, especially in the case of those with an irregular migratory status, make it difficult to access this therapy. Studies about the Mexico–United States border warn about the barriers undocumented migrants face in access to HIV prevention, detection and treatment due to their migrant condition [46].

The second moment corresponds to the SRH risks experienced during the migration process. Many of the young people interviewed arrived in Chile in recent years, where the context of the COVID-19 pandemic and the closure of borders drove an increase of entries through non-authorized points. These migratory patterns encourage violations of human rights during the trip, and survival strategies such as commercial sex work, which is highly risky for migrants, especially women and LGBTQI+ groups [47]. As Barot [48] argues, during migratory processes there is an increased threat to SRH that exposes, in particular, women and adolescent girls to unwanted pregnancy, unsafe abortion, STIs including HIV, and maternal illness and death. Hernandez et al. [46] propose the concept of “risk contexts” referring to situations in which structural variables such as poverty, lack of access to health systems and human trafficking create favorable conditions for serious violations of SRH rights of migrants, with consequent exposure to adverse health conditions such as the acquisition of STIs [29]. To date, most research and public policies have focused mainly on maternal and child health care for migrant women, ignoring the importance of the aforementioned issues for addressing the sexual health of specific migrant groups such as LGBTQI+ people, young women and adolescents.

The third moment refers to the settlement process in Chile. Although migrants correspond to a highly diverse and heterogeneous group, both because of their countries of origin, the conditions in which they carry out the migratory process, socioeconomic level, gender, ethnicity and age, among other variables [49], there is an international consensus to recognize immigration as a broad social determinant of health and wellbeing [50]. Although the migratory experience has an impact on the health of all migrants, health problems tend to be concentrated in those migrants who experience higher levels of poverty, social exclusion and discrimination [12]. This is the case, for example, of young Afro-descendant migrants who experience the racialization and sexualization of their bodies [51,52], a process that results of the study also show. Fanon accounts for the effects of racialized practices, which turn “non-white” bodies into objects of the hostile white gaze [53]. This white gaze operates as a strategy of power that enacts white supremacy and marks non-white bodies, which are subject to stigma and discrimination.

Postcolonial theory explains that this responds to the racial hierarchy of the “white” that has remained at the base of social inequalities in Latin America [54,55]. It is a cultural matrix that configures “superior” and “inferior” citizens, the bodies of black women being those who suffer the crudest processes of exclusion and discrimination through the hyper-sexualization of their bodies and their conceptualization as objects of pleasure [56,57].

Finally, the fourth moment has to do with the experiences of young migrants with the health system in Chile, in particular with SRH services. The results of the study show that for most of the young migrants interviewed, health is not a priority, but the priority is to survive. For the same reason, many say they are unaware of the particularities and functioning of the Chilean health system. A study carried out in Santiago de Chile showed that 50% of the adolescents and young migrants surveyed had not used the health system in the country, 60% did not know if they were formally enrolled in the health system, and 25% indicated not being enrolled [58]. In this regard, it is important to highlight that guaranteeing information on the functioning of the health system and access to biomedical services is not enough to ensure the real exercise of SRH rights of migrants. In fact, as confirmed in this research, health systems, despite the recommendations emanating from the Cairo Conference [59], have not yet separated sexual health from reproductive health, and the latter continues to be the priority in the approach to women’s health. By subsuming sexual health to reproductive health, health conditions such as STIs, the effects of sexual violence during migratory trajectories and other relevant issues are made invisible, although they have a great impact on the lives of young migrant populations. Furthermore, this research reveals the problems that the risk-focused approach, which persists in the specialized literature [19], has when dealing with the SRH of young migrants. As mentioned before, this is an approach that tends to focus attention on behavioral and cultural aspects of young migrants, hiding the barriers the social and health system itself places to the promotion of their wellbeing [20].

The study reveals that in their mobility experiences, migrant populations, and in particular young people, cross not only material borders but also symbolic, social and hierarchical ones. They organize networks, and mobilize affections and sexualities, all issues that are barely visible in classical approaches to young people’s migration processes [24,25,26]. In this sense, we adhere to the sexual turn in mobility studies; considering emotionality and sexuality as dimensions that need to be approached in migration studies [45], and that have not been addressed from the health perspective of migration, despite the obvious implications they have for the exercise of the right to health of populations in mobility [60,61]. Studies have investigated migrants’ and refugees’ health and quality of life mainly from a quantitative approach, and giving little attention to the latter dimensions [62].

To the best of our knowledge, this is one of very few studies in Chile and the broader region that explore the SRH vulnerabilities and inequities experienced by young migrant populations during their migration process and in the receiving country, and it therefore addresses a research gap. The study presents important insights for the improvement of the quality of SRH services for young migrant populations.

The study findings should be read with some limitations in mind. Data collection was beginning to be carried out when the COVID-19 pandemic struck, which imposed research restrictions we had not anticipated. A methodology that had been planned as ethnographical had to be re-designed to carrying out in-depth interviews with limited interaction of the research team in the study settings. Another limitation caused by the pandemic was the extension of fieldwork from the initially planned schedule, which implied greater difficulties in the recruitment of cases and in the continuity of the participation of the people initially contacted. Migrants constitute a hard-to-reach population, which was aggravated by the context of the pandemic, and additionally because more restrictive migration policies were implemented during this period. The COVID-19 pandemic also aggravated problems of health care access in general, and of SRH services in particular, for which the results of the study show a particular context and moment in time. Despite the particularity of this situation, it can shed important light on critical points of health services and ways to be better prepared for future health crises.

## 5. Conclusions

In this article, we focus on some aspects of SRH in the migration process of Latin American youth. The study shows the SRH risks to which young people are exposed during the migratory journey and during their settlement process in the host country, Chile: violence, sexual abuse, lack of documentation, poor access to information and discrimination within the health system. The results of the study emphasize the need to overcome the risk approach and primary focus on maternal health, which still persist in the study of young migrants’ health. Sexual health must be addressed as a central dimension of the lived experiences of young migrants. The social, cultural and structural factors that undermine SRH must be addressed.

## Figures and Tables

**Table 1:** Summary of main study findings.

Motivations to Migrate
Economic precarity Political instability Motivations related to SRH: ○ Pregnant women seeking better pregnancy and childbirth health care than in their home countries ○ LGBTQI+ people seeking social contexts of greater acceptance of gender and sexual diversity ○ HIV-positive people seeking access to treatment
Sexual and reproductive health risks during the migration process
Sexual abuse/rape Sex as a survival strategy: sexual exchange for money, transport, food and other subsistence itemsYoung women and the LGBTQI+ population at greatest risk
Settlement in Chile: Experiencing vulnerabilities
Social vulnerabilities in the settlement process, precarious housing and access to primary services Stereotypes, stigma and discrimination: ○ Young migrants perceive that the local population feels threatened by their presence in the country, and that they come to take away social benefits, including access to health ○ Association of afro-descendant bodies with exacerbated sexuality and commercial sex work ○ Stereotypes of Venezuelan and Colombian youth as engaged in gangs and drug trafficking
Health system in Chile: Sexual and reproductive health services
Health guarantees for the migrant population in decline and/or not always respected ○ Health staff perceives that the country’s new migration law has generated significant setbacks in migrants’ health guarantees ○ Lack of training and updating of health workers on regulations for access of migrants to the health system, and health rights of migrants Young migrants have a poor understanding of how the Chilean health system works: questions on requirements to access health care and whether it is free or not Administrative barriers as obstacles to access the health system ○ Accentuated in migrants in an irregular situation, without provisional or permanent ID ○ This is critical for HIV+ people who find barriers in accessing antiretroviral therapy, and for trans people who are denied protocols of recognition of their gender identities Stigma and discrimination towards migrants of the general society reproduced within the health sector. SRH services: ○ Pregnancy and childbirth-related issues as the most sought health care services by young migrants; generalized perception in migrants that young woman can suffer obstetric violence from health staff. Prompt access to contraceptives and condoms in primary care centers is highly valued

## Data Availability

Not available.
